# The Impact of Overweight Among Children on Salivary Vitamin D, Calcium, and Magnesium in Relation to Dental Caries Severity

**DOI:** 10.1155/ijod/4220296

**Published:** 2025-11-09

**Authors:** Shahba'a Munther, Hiba N. Yassin, Baydaa Hussain Awn

**Affiliations:** Department of Pediatric and Preventive Dentistry, College of Dentistry, University of Baghdad, Baghdad, Iraq

**Keywords:** dental caries severity, overweight, salivary calcium, salivary magnesium, salivary vitamin D

## Abstract

Vitamin D is one of several nutrients essential for calcium metabolism. Body weight status and magnesium may influence vitamin D activity. To determine whether salivary vitamin D, magnesium, and calcium levels are associated with body weight status and dental caries severity in children, this cross-sectional research was conducted.

**Methods:**

The sample consisted of 180 boys aged 6–8 years. According to their body mass index (BMI), children were assigned to three groups of 60 boys (normal weight, overweight, and obese). Moreover, within each weight group, the sample was divided into three groups according to caries severity (20 children in each group): mild (dmft ≤ 3), moderate (dmft = 4–6), and severe (dmft ≥ 7). Unstimulated whole saliva was obtained from each child in the morning (9 : 00–11 : 00 a.m.) at least 1 h after food or drink intake. Participants were seated and asked to accumulate saliva in the floor of their mouth and then spit into sterile, prelabeled polypropylene tubes over a 5 min period; the samples were then analyzed to assess salivary vitamin D, calcium, and magnesium concentrations.

**Results:**

Salivary vitamin D, calcium, and magnesium concentrations were significantly higher in boys with normal weight than in overweight and obese boys (*p* ≤ 0.05), the same results were recorded in mild caries children compared to those with moderate and severe caries (*p* ≤ 0.05). Interactions between BMI and caries severity in vitamin D, magnesium, and calcium were found that reached significant levels.

**Conclusion:**

This study provides preliminary evidence of associations between salivary (vitamin D, calcium, and magnesium) levels and both dental caries and overweight in boys. Given the cross-sectional design, limited sample size, and homogeneous population, the results should be interpreted with caution. Longitudinal studies are required to validate these biomarkers for routine clinical use.

## 1. Introduction

Obesity during childhood is a worldwide health problem that influences physical, mental, emotional, and social functions, and exposes the child to a wide range of health complications such as heart disease, diabetes mellitus (type II), sleep disorders, osteoarthritis, chronic inflammation, and increased oxidative stress [1–4]. Literatures recorded associations between both overweight and obesity and deficiency of vitamin D. Vitamin D is fat-soluble in nature, excess fat tissue can trap it and reducing its availability in the bloodstream. In addition to that, large body size increases the volume in which the vitamin distributed, leading to lower levels in blood. Furthermore, people with obesity may spend less time outdoors and have less sun exposure, limiting natural vitamin D production [5, 6]. On the other hand, vitamin D regulates hormones like leptin and insulin, deficiency of this vitamin disrupt these hormones' function, increasing appetite and storage. In addition, vitamin D deficiency impairs fat oxidation, promoting weight gain [7, 8]. Calcium and magnesium are essential cofactors in vitamin D activation; on the other hand, vitamin D promotes intestinal absorption of both calcium and magnesium. This interdependence indicated that vitamin D deficiency reduces calcium and magnesium bioavailability, and calcium or magnesium deficiency hinders the activation and function of vitamin D [9, 10]. Recently, there have been increasing concerns about the interaction between vitamin D and oral health during development and adulthood; a wide range of oral disease conditions are associated with low vitamin D [11]. During childhood, severe vitamin D deficiency can cause defective tooth mineralization, resulting in enamel and dentin defects, this can increase the beginning and progression of dental caries [12, 13]. Magnesium is a cofactor of vitamin D which helps vitamin D bind to its target proteins by controlling renal and hepatic vitamin D metabolism. Thus, without magnesium, vitamin D will not be useful, but it will be stored in the body [9, 14]. In addition to that, magnesium enhances enamel structure, plays a crucial role in remineralization against demineralization of early caries, and reduces cariogenic bacteria [15]. Right now, the gap is clear but could be sharpened. Stress that although serum vitamin D has been studied, salivary biomarkers are understudied and may provide a noninvasive alternative. This study was conducted to evaluate whether weight status among boys aged 6–8 years affects dental health status and salivary vitamin D, calcium, and magnesium. This study focused on boys to get a homogeneous sample and minimize the potential confounding factors related to genders that could allow clearer results; however, this limits the generalizability of results.

## 2. Methods

### 2.1. Study Sample

This cross-sectional study involved 180 boys aged 6–8 years, selected randomly from patients visiting dental clinic of the Dental College/University of Baghdad. They were healthy with no history of systemic diseases and chronic illness, and they were not taking medicines or supplements according to their parents' reports and the medical department in their school. Any boy did not meet this criteria was excluded from the study. This work was approved by the Ethics Committee of the College of Dentistry, University of Baghdad, (675322) is the approval number. Informed consents from children's parents/guardians have been taken.

G power software was applied previously to determine the number of participants required for this work. Depending on the probability alpha error = 0.05, effect size *F* = 0.40 (large effect size), number of groups = 9, the sample size was 135 participants, by adding 10% of error rate that equals 149 participants is enough to be involved in this work. Although 149 participants were required, 180 were included to increase power and account for potential dropouts.

### 2.2. Body Mass Index (BMI) Classification

Weight and height measurement was obtained from each study participant, and the BMI was determined (weight Kg/height m^2^). According to BMI, boys were classified into three groups of 60 boys (overweight, obese, and normal weight [control]). BMI classification was done according to the World Health Organization (WHO) growth standards for age and gender [16] as follows:

• Normal weight: BMI ranged from 5th percentile to 85th percentile
• Overweight: BMI ranged from 85th to 95th percentile
• Obese: BMI ≥ 95th percentile.

### 2.3. Dental Caries Assessment

**Figure 1:**
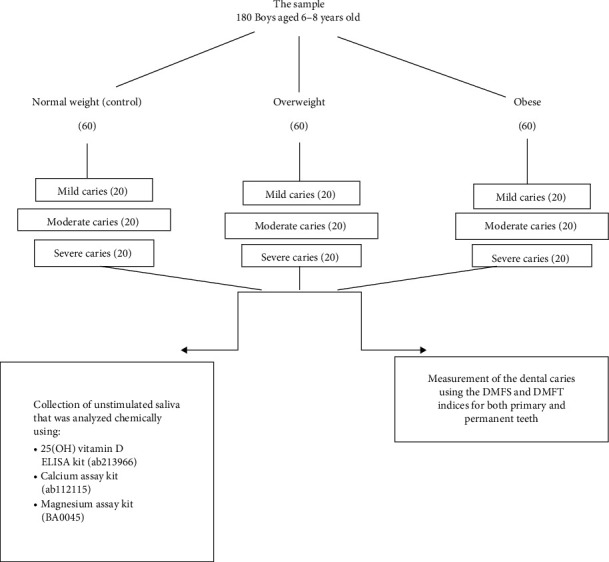
Design of the study.

The DMFT/dmft index was used to assess dental caries according to WHO criteria [17]. Two calibrated and trained examiners performed all clinical examinations. The agreement between the two examiners was assessed using Cohen's kappa before the start of work, it was found to be *κ* = 0.78, indicating substantial agreement. Each weight group was further classified into three subgroups (20 participants) according to caries severity [18]: mild ≤ 3, moderate (4–6), and severe ≥ 7. The study design is demonstrated in Figure 1.

### 2.4. Saliva Collection

Unstimulated whole saliva samples were collected according to the described method by Tenovuo and Lagerlöf [19]. To minimize the effect of circadian variability on the samples, saliva was sampled between 9 : 00 and 11 : 00 a.m. at least 1 h after food or drink intake. Participants were seated and asked to accumulate saliva in the floor of their mouth and then to spit into sterile, prelabeled polypropylene tubes over a 5 min period. Salivary samples were centrifuged for 10 min at 3000 rpm to remove cellular debris, the supernatant was then stored at −20°C for further biochemical assays. All steps were according to uniform handling protocols to ensure the accuracy and reliability of biochemical analysis and reliability and minimize preanalytical variability.

Salivary vitamin D, calcium, and magnesium were determined colourimetrically using a vitamin D ELISA kit (ab213966) for salivary vitamin D; this assay is a competitive, and has a detection range of approximately 0.5–1010 ng/mL. Intra-assay precision (repeatability) was good: coefficients of variation (CVs) were approximately 1.6% at high concentrations (~225 ng/mL), approximately 2.1% at mid (~37 ng/mL), and approximately 3.4% at low concentrations (~5 ng/mL). Interassay variability was higher, especially at mid-levels (~15.8% CV). All the procedures, including sample dilution, assay incubation, washing, and reagent storage, were carried out according to the manufacturer′s instructions to optimize reliability.

Calcium assay kit (ab112115) was used for the determination of salivary calcium levels; this assay is based on a red-fluorescent dye that binds specifically to free calcium ions to produce a fluorescence signal that is proportional to calcium concentration (mg/dL) (Ex/Em = 540/590 nm), with high sensitivity and little interference from other divalent cations. All reagents were stored per manufacturer's instructions (including protection of light-sensitive components), blacks/subtraction were applied to account for background fluorescence. Although manufacturer reports indicate good performance across the detection range, detailed precision (intra-assay and interassay CV) data were not publicly available at the time of this study.

Salivary magnesium was determined using magnesium assay kit (BA0045); the assay is based on calmagite dye. The dye forms a colored complex with Mg^2+^ in alkaline media. The measurement was taken at 500 nm using a microplate reader. The detection limit of the assay is 0.1 mg/dL, and the assay time is approximately 10 min. While specific intra-inter assay CV values were not provided by manufacturer, the assay's design and performance characteristics suggest a reliable measurement for salivary magnesium concentrations.

### 2.5. Data Analysis

SPSS software (version 21, IBM Corp., Armonk, NY, USA) was used for analysis of data. To check normality and determine the appropriate statistical tests, Shapiro–Wilk test was used. Descriptive statistics were applied, including: means ± standard deviations and counts. To evaluate the effect of each BMI category (normal weight, overweight, and obese) and caries severity (mild, moderate, and severe) separately on the studied variables (salivary vitamin D, calcium, and magnesium levels), a one-way multivariate analysis of variance (MANOVA) was performed. If the MANOVA results were statistically significant, follow-up one-way ANOVA tests were conducted for each dependent variable separately. To evaluate the combined effect and interaction between both BMI categories (normal weight, overweight, and obese) and dental caries severity (mild, moderate, and severe) together on the studied variables (salivary vitamin D, calcium, and magnesium levels), a two-way MANOVA was applied, and if its results was significant, two-way ANOVA tests were applied. Prior to conducting MANOVA, the assumption of multivariate normality, homogeneity of covariance matrices (Box's M test), and absence of multicollinearity were evaluated and found to be satisfied. Multiple comparisons were adjusted using Bonferroni correction to avoid the risk of type I error. To evaluate the bivariate correlations between salivary vitamin D, calcium, magnesium, and dental caries scores, Pearson's correlation coefficient was applied, *p* ≤ 0.05 was accepted as the statistical significance level.

## 3. Results

Shapiro–Wilk test indicated that the entire variable (salivary vitamin D, calcium, magnesium, and dental caries) were normally distributed (*p* ≤ 0.05) and met the requirements of parametric tests. A total of 180 boys were involved in this study, with 60 participants in each BMI group (normal weight, overweight, and obese) divided into three groups of 20 participants with in each weight group according to caries severity (mild, moderate, and severe). All participants completed saliva collection and caries assessment, and no data were excluded from the analysis.

Higher salivary vitamin D levels were recorded in normal weight boys, followed by overweight, but it was significantly lower in obese (8.078 ± 0.058, 7.989 ± 0.029, and 7.874 ± 0.023) ng/mL, respectively. Calcium and magnesium distributions were in the same pattern. The means were (50.634 ± 0.065, 50.404 ± 0.069, and 48.146 ± 1.612) mg/dL for calcium and (1.535 ± 0.034, 1.482 ± 0.015, and 1.448 ± 0.013) mg/dL for magnesium. One-way analysis of variance identified notable differences across the weight groups for the three parameters (vitamin D, calcium, and magnesium) (*p* ≤ 0.001 and *p* ≤ 0.01). Bonferroni-adjusted post hoc test indicated that each group was significantly different from the other two groups in all parameters (Table 1).

Concerning the caries severity, higher salivary vitamin D levels were noted in cases of mild caries, with subsequent decrease in moderate cases, then in severe caries cases (8.031 ± 0.127, 7.960 ± 0.087, and 7.949 ± 0.093) ng/mL, respectively. Calcium and magnesium showed the same pattern among the caries groups. The salivary calcium levels were (50.506 ± 0.206, 49.561 ± 1.616, and 49.112 ± 2.323) mg/dL and salivary magnesium levels were (1.513 ± 0.064, 1.484 ± 0.025, and 1.470 ± 0.045) mg/dL, respectively. The results of one-way ANOVA test demonstrated significant variance among the caries groups for the three parameters (vitamin D, calcium, and magnesium) (*p* ≤ 0.001 and *p* ≤ 0.01). Bonferroni test of post hoc component indicated that each group was significantly different from the other two groups in all studied variables (Table 2).

The three studied variables varied according to both the weight status and caries severity of the participants. Among normal-weight participants, vitamin D levels in saliva slightly declined with increasing caries severity, in mild (8.159 ± 0.096 ng/mL), in moderate (8.038 ± 0.212 ng/mL), and in severe (8.036 ± 0.188 ng/mL). Calcium and magnesium showed a similar pattern. Calcium levels in saliva decreased slightly from mild caries (50.718 ± 0.075 mg/dL) to severe cases (50.559 ± 0.087 mg/dL). Magnesium levels also showed a small reduction from 1.583 ± 0.067 to 1.519 ± 0.046 mg/dL. In overweight children, vitamin D levels also showed a downward trend with caries severity, in mild cases (8.029 ± 0.182 ng/mL), in moderate (7.977 ± 0.205 ng/mL), and in severe cases (7.961 ± 0.217 ng/mL). A similar gradual decrease was observed for calcium and magnesium. Calcium levels declined from 50.495 ± 0.067 mg/dL (mild) to 50.345 ± 0.088 mg/dL (severe), and magnesium from 1.496 ± 0.049 (mild) to 1.462 ± 0.062 mg/dL (severe). Among obese participants, the decline was more pronounced in vitamin D from 7.906 ± 0.182 ng/mL (mild) to 7.851 ± 0.208 ng/mL (severe), calcium from 50.306 ± 0.121 mg/dL (mild) to 46.433 ± 4.775 mg/dL (severe) and magnesium from 1.459 ± 0.066 (mild) to 1.430 ± 0.066 mg/dL (severe) (Table 3).

A significant main effect was recorded between both weight status and caries severity on all salivary variables (vitamin D, calcium, and magnesium) as shown by two-way ANOVA. Weight status significantly affects salivary vitamin D, calcium, and magnesium (*F* = 7.620, 18.732, and 7.643), respectively (*p* ≤ 0.001). Similarly, caries severity showed significant effects on vitamin D, calcium, and magnesium (*F* = 4.565, 4.919, and 19.975), respectively, (*p* ≤ 0.001). The interaction effects between weight status and caries severity were also significant for all variables: vitamin D (*F* = 0.600, *p* ≤ 0.05), calcium (*F* = 4.016, *p* ≤ 0.01), and magnesium (*F* = 2.548, *p* ≤ 0.05). Furthermore, the two-way MANOVA indicated a statistically significant interaction effect of weight status and caries severity on the studied salivary variables (Wilks' lambda = 0.793, *F* = 2.501, *p* ≤ 0.01), suggesting a multivariate difference in salivary profiles depending on both factors (Table 4).

The studied salivary biomarkers recorded negative correlations with both dental caries severity and BMI that reached a significant level. Negative correlation was recorded between salivary vitamin D levels and both caries severity (*r* = −0.385, *p* ≤ 0.001) and BMI (*r* = −0.299, *p* ≤ 0.01). Conversely, salivary vitamin D recorded a positive correlation with both salivary calcium (*r* = + 0.271, *p* ≤ 0.01) and magnesium (*r* = + 0.230, *p* ≤ 0.01).Calcium levels were also negatively correlated with both caries severity (*r* = −0.193, *p* ≤ 0.05) and BMI (*r* = −0.185, *p* ≤ 0.05), whereas they showed a positive correlation with magnesium (*r* = + 0.190, *p* ≤ 0.05). Salivary magnesium levels were negatively correlated with both caries severity (*r* = −0.485, *p* ≤ 0.001) and BMI (*r* = −0.392, *p* ≤ 0.01) (Table 5).

The result section focused on salivary mineral levels, BMI, and caries severity. Potential confounding factors such as dietary habits, socioeconomic status, salivary flow rate, and oral hygiene practices were not determined in this study.

## 4. Discussion

Vitamin D is a lipophilic micronutrient with several important roles in the body, including calcium-phosphate metabolism, immune system regulation, bone development, and more. The body mass- vitamin D association had been explored by various studies, with many reporting an inverse association between the two [7, 8]. However, regarding salivary vitamin D and its potential relationship with body weight, there are fewer studies on this topic. As such, the target of this work was to identify the interaction between salivary vitamin D, calcium, and magnesium with body weight and caries severity. Only boys were involved in this cross-sectional research to maintain the homogeneity of the population and increase the internal validity by removing gender as a possible source of variation.

Salivary vitamin D, calcium, and magnesium were higher in normal weight boys and lower in obese children. Furthermore, significant negative correlations were recorded between salivary vitamin D, magnesium, and calcium levels and BMI. Previous research has repeatedly confirmed BMI-serum vitamin D association [11, 20, 21]. However, there are also few studies that failed to record such an association [22]. The possible mechanisms of decreased vitamin D in obese individuals include sequestration of the vitamin in fat depots and impaired synthesis in adipose tissue and hepatocytes [23, 24]. Magnesium deficiency has also been found to be related to consumption of an unhealthy carbohydrate-based diet. Magnesium is responsible for the vitamin D bioactivation that occurs in the liver and kidneys [25, 26]. As such, magnesium deficiency in obese individuals may result in vitamin D deficiency. As a result, the interaction and negative correlations recorded in the present work between the measured variables can be explained by the recorded vitamin D-magnesium correlation.

Concentrations of the studied nutrients were also elevated in mild caries and decreased in severe cases. In addition to that, these nutrients correlated negatively with dental caries with significant levels. Calcium protective role against caries is well-known in the literature, mostly through its role in the remineralization process by the formation of hydroxyapatite crystals [27]. Calcium also requires vitamin D for its absorption and to be available in the serum and other tissues. The presence of vitamin D and calcium deficiencies, therefore, may impair the ability of enamel and teeth to fight caries. The results obtained by this study regarding the impact of salivary vitamin D and calcium on the dental caries are in line with a number of previous studies [28–30], although the literature also includes some contradictory results [31, 32]. As for the results of the effect of salivary magnesium on the development of caries, these were in agreement with one study [33], but in disagreement with another [34]. The possible explanation of caries reduction among those with adequate salivary magnesium levels is that magnesium is a natural component of the hydroxyapatite crystals that enhances enamel mineralization and stability. Furthermore, salivary magnesium enhances salivary buffer capacity, thus reduces demineralization [15]. The positive intercorrelations and the significant interaction between all measured variables that were obtained by this study support the hypothesis of the synergistic relationship between them.

In terms of clinical significance, according to the present findings, the concentrations of salivary vitamin D, calcium, and magnesium could be used as an effective tool for assessing the risk for dental caries and the nutritional status of pediatric patients, especially among individuals at risk for obesity and malnutrition.

However, the study has several limitations that should be reported. The relatively small size of the sample and the homogeneous study population, including only boys, limit the generalizability of the finding to abrader and more diverse populations. Potential confounding factors, including diet, stress levels, hygiene behavior, socioeconomic status, and exposure to sun, were not fully controlled and may affect biomarker concentrations. The cross-sectional design prohibits causal inference, and the observed relationship should be interpreted as associative rather than evidence of cause-and-effect. These limitations indicated that the present findings should be regarded as preliminary, underscoring the need for larger, longitudinal studies to validate and extend these results.

Future research could investigate both genders to achieve comprehensive knowledge about the interaction between the studied variables and caries development in children and to allow for the generalisability of the results in populations at risk for obesity and malnutrition. A longitudinal or interventional study design is also recommended to explore the possible causal relationship between the measured variables. Future studies should also consider other possible variables such as diet, hygiene behavior, socioeconomic status, and exposure to sun.

## 5. Conclusion

This study provides preliminary evidence of associations between salivary (vitamin D, calcium, and magnesium) levels and both dental caries and overweight in boys. Given the cross-sectional design, limited sample size, and homogeneous population, the results should be interpreted with caution. Longitudinal studies are required to validate these biomarkers for routine clinical use.

## Figures and Tables

**Table 1:** Salivary concentrations and statistical differences (one-way ANOVA) of vitamin D, calcium, and magnesium among children grouped by BMI.

Variable	Normal weight (*n* = 60)	Overweight (*n* = 60)	Obese (*n* = 60)	One-way ANOVA	
	Mean ± SD	Mean ± SD	Mean ± SD	*F*-test	*p*-Value
Vitamin D (ng/mL)	8.078 ± 0.058	7.989 ± 0.029	7.874 ± 0.023	7.620	≤ 0.01
Calcium (mg/dL)	50.634 ± 0.065	50.404 ± 0.069	48.146 ± 1.612	16.278	≤ 0.001
Magnesium (mg/dL)	1.535 ± 0.034	1.482 ± 0.015	1.448 ± 0.013	5.728	≤ 0.01

Abbreviations: dL, deciliter; mg, milligram; mL, milliliter; ng, nanogram; No., number; SD, standard deviation.

**Table 2:** Salivary concentrations and statistical differences (one-way ANOVA) of vitamin D, calcium, and magnesium among children grouped by caries severity.

Variable	Caries severity			One-way ANOVA	
	Mild (*n* = 60)	Moderate (*n* = 60)	Severe (*n* = 60)		
	Mean ± SD	Mean ± SD	Mean ± SD	*F*-test	*p*-Value
Vitamin D (ng/mL)	8.031 ± 0.127	7.960 ± 0.087	7.949 ± 0.093	4.147	≤ 0.05
Calcium (mg/dL)	50.506 ± 0.206	49.561 ± 1.616	49.112 ± 2.323	3.617	≤ 0.05
Magnesium (mg/dL)	1.513 ± 0.064	1.484 ± 0.025	1.470 ± 0.045	17.406	≤ 0.001

Abbreviations: dL, deciliter; mg, milligram; mL, milliliter; ng, nanogram; No., number; SD:, standard deviation.

**Table 3:** Salivary concentrations of vitamin D, calcium, and magnesium among children grouped by BMI and dental caries severity.

Caries severity	Weight status	Vitamin D (ng/mL)	Calcium (mg/dL)	Magnesium (mg/dL)
Mild	Normal	8.159 ± 0.096	50.718 ± 0.075	1.583 ± 0.067
	Overweight	8.029 ± 0.182	50.495 ± 0.067	1.496 ± 0.049
	Obese	7.906 ± 0.182	50.306 ± 0.121	1.459 ± 0.066

Moderate	Normal	8.038 ± 0.212	50.625 ± 0.093	1.505 ± 0.061
	Overweight	7.977 ± 0.205	50.357 ± 0.058	1.490 ± 0.055
	Obese	7.866 ± 0.271	47.701 ± 4.233	1.456 ± 0.068

Severe	Normal	8.036 ± 0.188	50.559 ± 0.087	1.519 ± 0.046
	Overweight	7.961 ± 0.217	50.345 ± 0.088	1.462 ± 0.062
	Obese	7.851 ± 0.208	46.433 ± 4.775	1.430 ± 0.066

*Note: n* = 20 per cell.

Abbreviations: dL, deciliter; mg, milligram; mL, milliliter; ng, nanogram; SD, standard deviation.

**Table 4:** The differences and interactions among salivary variables by weight and caries severity.

Two-way ANOVA test			
Variable	Source	*F*-test	*p*-Value
Vitamin D (ng/mL)	Weight	7.620	≤ 0.01
	Caries	4.565	≤ 0.05
	Weight × caries	0.600	≤ 0.05

Calcium (mg/dL)	Weight	18.732	≤ 0.001
	Caries	4.919	≤ 0.01
	Weight × caries	4.016	≤ 0.01

Magnesium (mg/dL)	Weight	7.643	≤ 0.01
	Caries	19.975	≤ 0.001
	Weight × caries	2.548	≤ 0.05

**Two-way MANOVA-test**			
**Weight × caries**			
**Salivary variables**	***F*-test**	**Sig.**	**Wilk's lambda**

Vitamin D (ng/mL)	2.501	*p* ≤ 0.01	0.793
Calcium (mg/dL)			
Magnesium (mg/dL)			

**Table 5:** Pearson correlation coefficients between salivary variables and dental caries severity, BMI, calcium, and magnesium.

Salivary variables	The study groups							
	Dental caries severity		Body mass index (BMI)		Calcium		Magnesium	
	*r*	*p*-Value	*r*	*p*-Value	*r*	*p*-Value	*r*	*p*-Value
Vitamin D (ng/mL)	−0.385^b^	≤ 0.001	− 0.299^b^	≤ 0.01	+0.271^b^	≤ 0.01	+0.230^b^	≤ 0.01
Calcium (mg/dL)	−0.193^a^	≤ 0.05	−0.185^a^	≤ 0.05	—	—	+0.190^a^	≤ 0.05
Magnesium (mg/dL)	−0.485^b^	≤ 0.01	−0.392^b^	≤ 0.01	—	—	—	—

^a^Significant.

^b^Highly significant.

## Data Availability

Anonymized data supporting the findings of this study are available from the corresponding author upon reasonable request.

## Nomenclature

WHO: World Health Organization
BMI: Body mass index.
